# Coptidis Rhizoma Water Extract Attenuates RANKL-Induced Osteoclast Differentiation via MAPK, Akt, and NF-κB Pathways and Prevents Ovariectomy (OVX)-Mediated Bone Loss

**DOI:** 10.3390/ijms26178707

**Published:** 2025-09-06

**Authors:** Sang-Yong Han, Yun-Kyung Kim

**Affiliations:** 1 Department of Herbal Medicine, College of Pharmacy, Wonkwang University, 460 Iksandae-ro, Iksan 54538, Republic of Korea; 030745@daum.net; 2 Wonkwang Oriental Medicines Research Institute, Wonkwang University, 460 Iksandae-ro, Iksan 54538, Republic of Korea

**Keywords:** *Coptidis rhizoma*, osteoclast differentiation, RANKL, ovariectomy, bone loss

## Abstract

Excessive osteoclast activity in bone remodeling can lead to an imbalance between bone resorption and formation, a common occurrence in abnormal bone metabolic diseases. This research investigates the effect of *Coptidis rhizoma* water extract (CRW) on osteoclastogenesis provoked by RANKL in vitro and bone destruction mediated by ovariectomy (OVX) in vivo. CRW, prepared from dried *Coptidis rhizoma* (CR), was analyzed for its active compounds—coptisine and berberine—using HPLC analysis. CRW markedly decreased the size and number of TRAP-positive multinucleated cells (TRAP^+^ MNCs), suppressed F-actin ring formation, and diminished bone resorption in RANKL-treated cultures. In the early phase of differentiation, CRW suppressed the phosphorylation of MAPKs p38, JNK, and ERK, as well as NF-κB p65, Iκ-Bα, and Akt. CRW also down-regulated RANKL-mediated induction of c-Fos and NFATc1 and attenuated the activation of NFATc1- dependent genes, such as *OSCAR*, *ATP6V0D2*, *ACP5* (TRAP), *OC-STAMP*, *DC-STAMP*, *CTSK* (cathepsin K), *CALCR* (calcitonin receptor), and *MMP-9*. In ovariectomized rats, micro-CT and histological analyses showed that CRW alleviated femoral bone destruction. These findings indicate that CRW restrains osteoclast differentiation and function and may have therapeutic potential for disorders driven by excessive osteoclast activity.

## 1. Introduction

The dynamic process of bone homeostasis ensures the preservation of skeletal mass and quality, simultaneously safeguarding organs and supporting hematopoiesis through continuous bone remodeling across an individual’s lifespan [1,2]. Remodeling proceeds as a coupled sequence in which osteoblasts lay down new bone and osteoclasts remove old bone, and these complementary activities normally occur in a tightly coordinated manner [1]. When this coupling is disturbed, metabolic diseases of the skeleton can emerge, such as osteoporosis, Paget’s disease, and periprosthetic osteolysis [3,4]. A common feature of these conditions is an increase in osteoclast function that drives excessive resorption and accelerates structural deterioration [5].

Formed through the fusion of mononuclear cells, osteoclasts are enormous multinucleated cells produced from hematopoietic stem cells within the monocyte–macrophage lineage, and they play a vital role in the intricate process of bone remodeling [6]. The differentiation process of osteoclasts is tightly controlled by various signaling pathways, including the receptor activator of the nuclear factor-κB ligand (RANKL) signaling pathway [7]. RANKL produced by osteoblasts engages the RANK receptor on the surface of osteoclast precursors and initiates downstream cascades that include Akt, NF-κB, and mitogen-activated protein kinases such as ERK, JNK, and p38 [7,8,9]. The initiation of these pathways ultimately leads to the induction of transcription factors like c-Fos and the nuclear factor of activated T-cells 1 (NFATc1), in turn promoting the expression levels of osteoclast-related marker genes essential for osteoclast differentiation and function, including cathepsin K (*CTSK*), tartrate-resistant acid phosphatase (*ACP5*, also known as TRAP), osteoclast-associated receptor (*OSCAR*), dendritic cell-specific transmembrane protein (*DC-STAMP*), osteoclast-stimulatory transmembrane protein (*OC-STAMP*), ATPase H+ transporting lysosomal 38kDa V0 subunit d2 (*ATP6V0D2*), matrix metallopeptidase-9 (*MMP-9*), and calcitonin receptor (*CALCR*) [10,11,12,13].

*Coptidis rhizoma* (CR) is a medicinal herb from the *Ranunculaceae* family. It has been used in East Asian traditional medicine to treat diarrhea, vomiting, high fever, diabetes, toothache, jaundice, coma, abdominal fullness, and eczema [14]. Numerous studies have shown that CR exhibits various pharmacological effects, including anti-hypertensive, anti-diabetic, anti-cancer, anti-inflammatory, anti-oxidative, cardioprotective, hepatoprotective activities, anti-Alzheimer, and neuroprotective [14,15,16,17,18,19]. More than 120 chemical compounds have been identified in CR, encompassing alkaloids, phenylpropanoids, lignans, flavonoids, saccharides, phenolic acids, and steroids [14]. Among these, protoberberine alkaloids such as berberine, coptisine, epiberberine, palmatine, columbamine, and jatrorrhizine are regarded as principal active components that mediate many of the observed bioactivities [20]. Recent studies have revealed that berberine and coptisine restrain osteoclast differentiation and suppress osteoclast function through mechanisms linked to MAPK signaling [21,22]. Based on these findings, we strongly suggest that Coptidis Rhizoma Water Extract (CRW) containing berberine and coptisine can inhibit RANKL-induced osteoclast differentiation. CRW contains various active ingredients in addition to berberine and coptisine, suggesting the possibility of synergistic and complementary effects through the interaction of these compounds [14], and so CRW would have a strong inhibitory effect on osteoclast differentiation. To confirm our hypothesis, we evaluated the inhibitory effects of CRW on the signaling pathways concerned in RANKL-stimulated osteoclastogenesis using mouse primary bone marrow-derived macrophages (BMMs) as a model of osteoclast precursors. In addition, we assessed the ameliorative effects of CRW in an ovariectomy (OVX)-associated bone loss animal model in SD rats by analyzing parameters of trabecular bone microarchitecture from Micro-CT.

## 2. Results

### 2.1. Determination of Coptisine and Berberine Concentration in CRW

**Figure 1:**
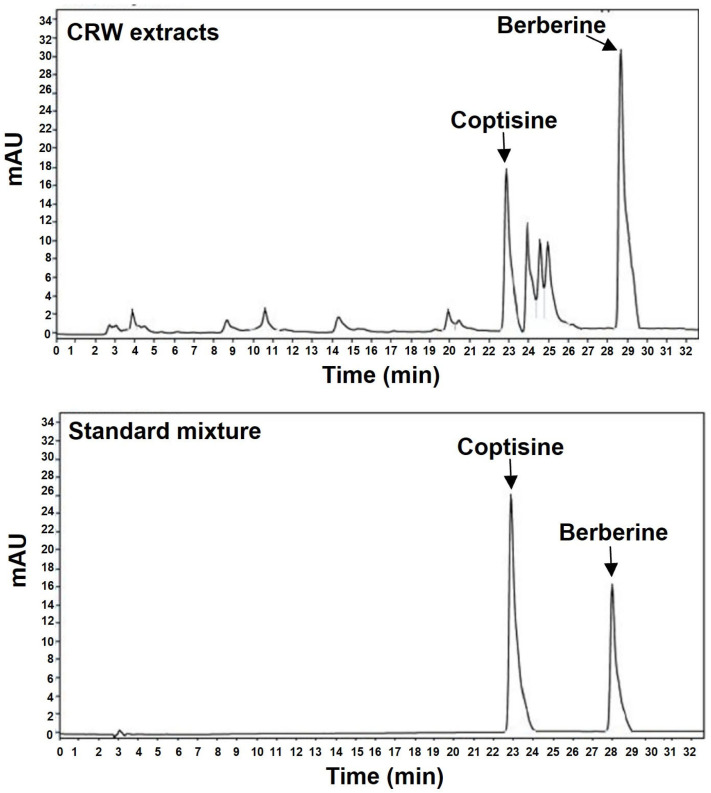
HPLC chromatograms of CRW (**upper**) and the standard coptisine and berberine (**lower**).

We first used HPLC to validate the existence of berberine and coptisine in CRW. As shown in Figure 1, we detected coptisine and berberine in CRW at the same retention time (RT) as that of the standard sample. The first peak (RT = 22.86 min) eluted by HPLC was identified as coptisine, and the second peak (RT = 27.96 min) was identified as berberine. The concentrations of berberine and coptisine in CRW were 26.1 mg/g and 5.7 mg/g, respectively.

### 2.2. CRW Blocks RANKL-Activated Osteoclast Differentiation Without Cytotoxicity

**Figure 2:**
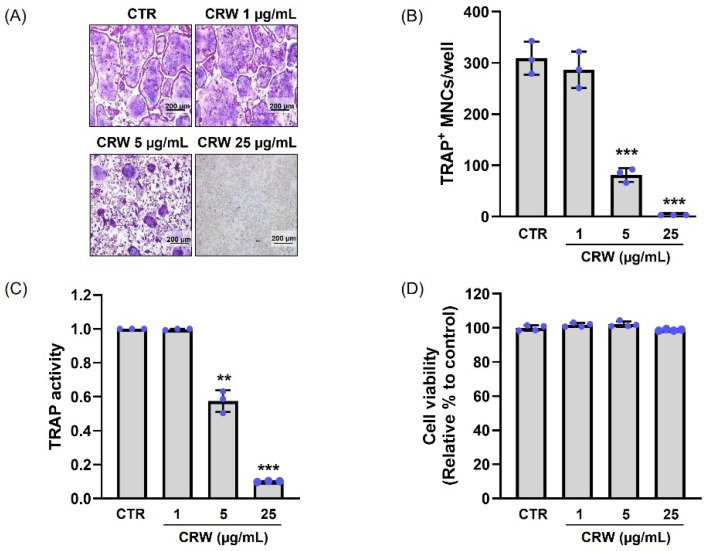
CRW decreased TRAP-positive osteoclast formation and enzymatic activity. BMMs were maintained for 4 days with CRW (1, 5, or 25 μg/mL) under M-CSF (30 ng/mL) and RANKL (100 ng/mL). (**A**) Images were taken using a microscope after cells were fixed and stained with TRAP solution. Scale bar = 200 μm. (**B**) Counts of TRAP^+^ multinucleated cells. (**C**) TRAP activity quantified as optical density at 450 nm. (**D**) Cell viability after 4 days of CRW exposure measured by the XTT assay. Blue dots represent individual values for each replicate within the group. ** *p* < 0.01 and *** *p* < 0.001 vs. control (CTR).

To investigate whether CRW restrains RANKL-stimulated osteoclastogenesis in the BMMs, we demonstrated the generation of TRAP-positive multinucleated cells (TRAP^+^ MNCs) and counted their number. BMMs were cultured with CRW (1, 5, and 25 μg/mL) for 4 days in α-MEM supplemented with M-CSF and RANKL. We confirmed the level of osteoclast differentiation using TRAP, which is a histochemical marker used to identify multinucleated osteoclasts. RANKL treatment increased the size of osteoclasts and the ratio of TRAP^+^ MNCs (Figure 2A). However, we observed that CRW prominently reduced the number of mature osteoclasts and TRAP^+^ MNCs, as shown in the images captured under the light microscope (Figure 2A). Interestingly, we observed a concentration-dependent decrease in the number of TRAP^+^ MNCs after CRW (Figure 2B). In particular, we determined that 25 μg/mL CRW considerably lowered the number of osteoclasts compared with the control (CRW-untreated group) (Figure 2B). Total TRAP activity measured across mono, di, and multinucleated populations was reduced by CRW relative to the RANKL control, confirming functional inhibition of osteoclastogenesis (Figure 2C). Subsequently, we treated BMMs with CRW for 4 days and evaluated their viability using the XTT assays. We found that compared with the control, neither of the tested CRW concentrations (1, 5, and 25 μg/mL) resulted in any cytotoxic effects after 4 days (Figure 2D).

Thus, we selected the CRW concentration of 25 μg/mL, which exhibited an effective inhibitory effect on RANKL-stimulated TRAP formation without cytotoxicity, for all subsequent experiments.

### 2.3. CRW Suppresses F-Actin Ring and Bone Resorption Activated by RANKL

**Figure 3:**
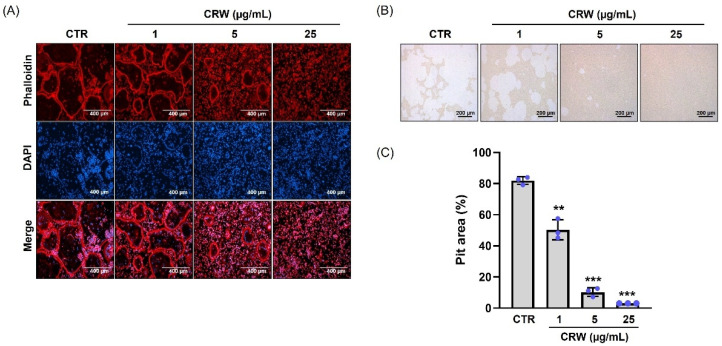
CRW mitigated RANKL-activated formation of F-actin rings and resorption pits. (**A**) BMMs were cultured with CRW (1, 5, or 25 μg/mL) under M-CSF and RANKL treatment for 4 days. BMMs were fixed in 3.7% formalin, and then permeabilized with 0.1% Triton X-100. F-actin was visualized with Texas-red phalloidin (Red), and nuclei were counterstained with DAPI (Blue). Scale bar = 400 μm. (**B**,**C**) Mature osteoclasts were seeded on hydroxyapatite coated plates with or without CRW. The adherent cells were detached and captured using a light microscope. Pit regions were assessed in each condition using ImageJ. Scale bar = 200 μm. Blue dots represent individual values for each replicate within the group. ** *p* < 0.01 and *** *p* < 0.001 vs. CTR.

We conducted immunocytochemistry staining to identify the effect of CRW on the F-actin ring formation in mature osteoclasts, a crucial structure in actively resorbing osteoclasts within BMMs [23]. As expected, robust F-actin rings appeared in the RANKL control, whereas CRW treatment diminished F-actin ring formation (Figure 3A). To decide whether CRW affects bone resorption activity, we cultured primary osteoblasts and BMCs on Osteo assay surface plates with growth medium containing prostaglandin E2 (PGE2) and 1,25-dihydroxyvitamin D3 (VitD3). RANKL increased the resorption activity of mature osteoclasts (Figure 3B). Conversely, treatment with 25 μg/mL CRW almost completely attenuated the RANKL-driven osteoclast resorption (Figure 3B). We also evidenced a significant decrease in total resorption pit area in CRW-exposed cells compared with RANKL-exposed cells (Figure 3C).

### 2.4. CRW Downregulates RANKL-Mediated Induction of NFATc1 and c-Fos

**Figure 4:**
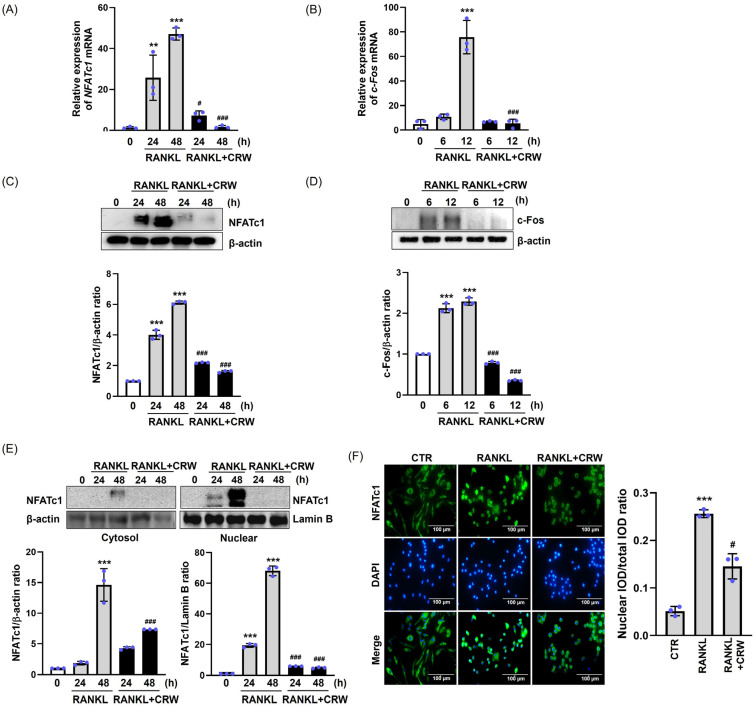
CRW suppresses RANKL-activated expression of NFATc1 and c-Fos. BMMs were pre-treated with or without 25 μg/mL CRW and then exposed to RANKL. (**A**,**B**) NFATc1 and c-Fos transcripts were quantified by qPCR. (**C**,**D**) Protein abundance of NFATc1 and c-Fos was examined by Western blotting with β-actin as the loading control. (**E**) Proteins extracted from the cytoplasmic and nuclear fractions were examined by Western blotting. Internal controls for nuclear and cytoplasmic proteins included Lamin B and β-actin, respectively. (**F**) Cells were fixed in 4% paraformaldehyde, permeabilized with 0.1% Triton X-100, and labeled with mouse monoclonal NFATc1 antibody (green) and DAPI (blue). Scale bar = 100 μm. ** *p* < 0.01, *** *p* < 0.001 vs. RANKL-untreated cells, ^#^ *p* < 0.05, ^###^ *p* < 0.001 vs. the RANKL-treated cells at the indicated times. Western blotting bands and NFATc1 nuclear/total integrated optical density (IOD) ratio were quantified using ImageJ. Blue dots represent individual values for each replicate within the group. Results are expressed as the mean ± S.D. of three independent experiments.

NFAFc1 and c-Fos transcriptional factors serve pivotal functions as modulators in osteoclastogenesis [24]. To confirm whether CRW suppresses the induction of NFATc1 and c-Fos during osteoclastogenesis triggered by RANKL, we conducted Western blotting and qPCR on CRW/RANKL-treated cells. As shown in Figure 4A,B, the mRNA levels of *NFATc1* (24–48 h) and *c-Fos* (6–12 h) were increased in response to RANKL at various time points, whereas it was significantly decreased by CRW treatment. Protein analyses supported these results. CRW markedly lowered RANKL-activated NFATc1 and c-Fos protein levels (Figure 4C,D). We next examined subcellular localization of NFATc1 during differentiation. Additionally, we found that RANKL activation increased the translocation of NFATc1 from the cytoplasm into the nucleus during osteoclast differentiation. We confirmed whether CRW had an inhibitory effect on the nuclear translocation of NFATc1 in osteoclastogenesis. As illustrated in Figure 4E, RANKL stimulation for 48 h increased nucleus NFATc1 expression relative to RANKL-untreated cells. Whereas, under the same conditions, CRW treatment inhibited the translocation of NFATc1 into the nucleus. Immunofluorescence staining further suggested enhanced translocation of NFATc1 into the nucleus, as evidenced by a notable elevation in colocalization and intensity of NFATc1 and DAPI in RANKL-only stimulated cells. Co-treatment with CRW and RANKL inhibited the NFATc1 nuclear translocation compared with RANKL alone, which is illustrated in Figure 4F.

### 2.5. CRW Attenuates RANKL-Activated Induction of Osteoclast Marker Genes

Activation of NFATc1 by RANKL drives late-stage transcriptional programs that support osteoclast differentiation and function. We next decided whether CRW had an inhibitory effect on the activation of osteoclast marker genes related to RANKL-associated osteoclast differentiation, by qPCR.

**Figure 5:**
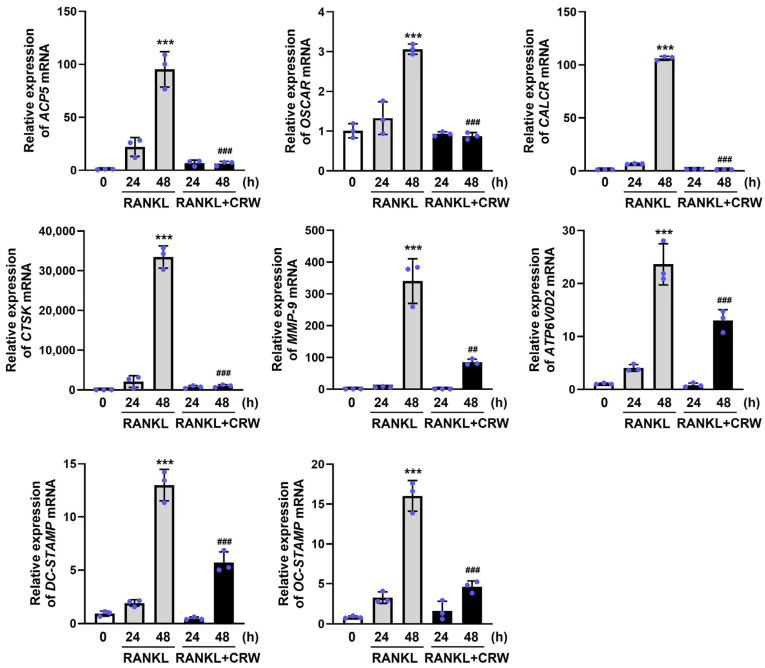
CRW downregulates RANKL-regulated osteoclast-related genes. BMMs were exposed to M-CSF and RANKL, either with or without 25 μg/mL CRW. mRNA expressions of *OSCAR*, *ATP6V0D2*, *ACP5*, *OC-STAMP*, *DC-STAMP*, *CTSK*, *CALCR*, and *MMP-9* were confirmed by qPCR. Measured data were normalized to GAPDH. Bars show mean ± SD from three independent experiments. Blue dots represent individual values for each replicate within the group. *** *p* < 0.001 vs. RANKL-untreated cells, ^##^ *p* < 0.01, ^###^ *p* < 0.001 vs. the RANKL-treated cells at the indicated times.

RANKL significantly upregulated mRNA levels of target genes involved in osteoclast function and differentiation, including *OSCAR*, *ATP6V0D2*, *ACP5*, *OC-STAMP*, *DC-STAMP*, *CTSK*, *CALCR*, and *MMP-9* (Figure 5). In contrast, CRW (25 μg/mL) efficiently inhibited the expression of these markers induced in the late stages of osteoclast differentiation by RANKL (Figure 5).

### 2.6. CRW Inhibited Early Signaling Pathway

**Figure 6:**
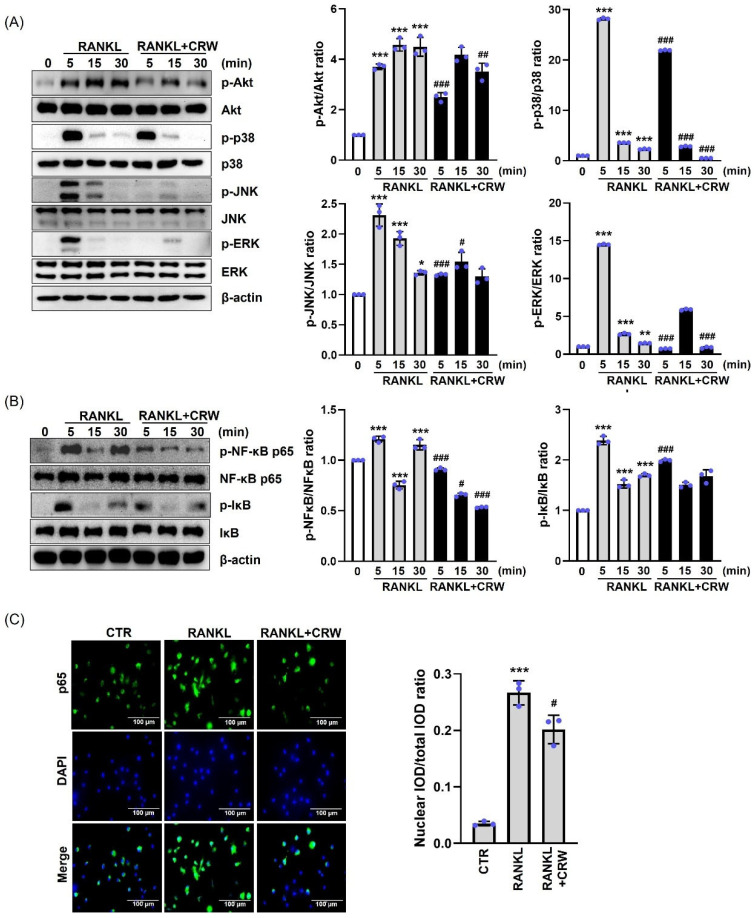
CRW reduces RANKL-mediated early-stage signaling. BMMs were pre-treated with or without 25 μg/mL CRW for 1 h before RANKL stimulation. (**A**,**B**) The total protein obtained from cells was analyzed using Western blot with the specified antibodies. (**C**) Immunofluorescent analysis. The cells were pigmented with anti-p65 antibodies (green) and DAPI (blue). Scale bar = 100 μm. * *p* < 0.05, ** *p* < 0.01 and *** *p* < 0.001 vs. RANKL-untreated cells, ^#^ *p* < 0.05, ^##^ *p* < 0.01, ^###^ *p* < 0.001 vs. the RANKL-stimulated cells at the indicated times. Western blot bands and p65 nuclear/total integrated optical density (IOD) ratio were quantified in ImageJ. Blue dots represent individual values for each replicate within the group. Results are expressed as the mean ± S.D. of three independent experiments.

To clarify the mechanistic role of CRW in osteoclast differentiation, we explored the inhibitory effects of CRW on the early signaling pathways provoked by RANKL. Our observations revealed that the phosphorylation of Akt, ERK, JNK, p38, and NF-kB peaked at about 5 min after RANKL exposure (Figure 6A,B). Treatment with CRW for 5 min remarkably diminished RANKL-activated phosphorylation of Akt, JNK, ERK, and p38, as demonstrated in Figure 6A. Upon RANKL stimulation, cytosolic IκB became phosphorylated and NF-κB p65 accumulated in the nucleus [25]. To further test how CRW influences NF-κB p65-dependent transcription, we performed Western blotting and immunocytochemistry analyses. We found that CRW prominently attenuated p-IκB and p-p65 in the NF-κB signaling pathway (Figure 6B). Moreover, CRW diminished the translocation of nuclear NF-κB p65 (Figure 6C).

### 2.7. CRW Recovered Bone Destruction in Ovariectomized Rats

**Figure 7:**
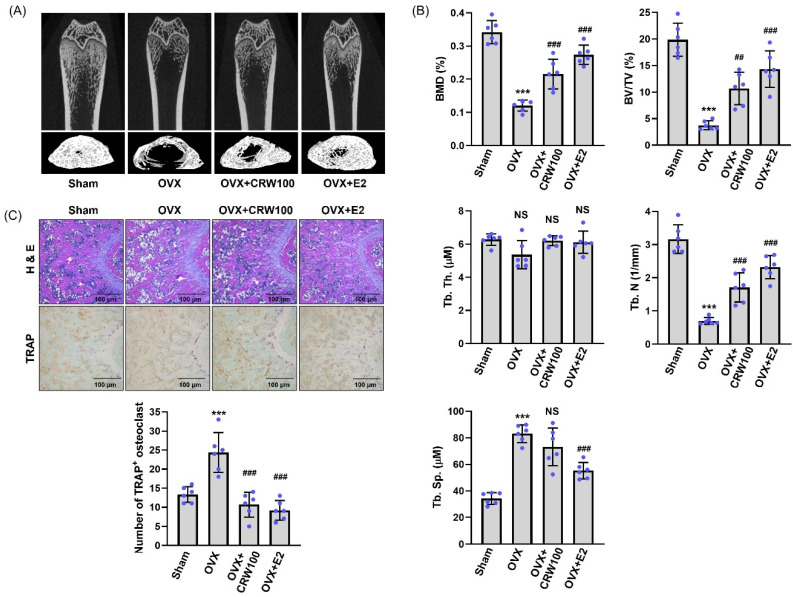
CRW restores OVX-mediated bone destruction. Female SD rats were bilaterally ovariectomized at 8 weeks. Animals received oral PBS and CRW (100 mg/kg) once daily for 12 weeks and were then euthanized. (**A**) Micro-CT was used to acquire images of longitudinal and transverse sections of trabecular bone. (**B**) Micro-CT data were analyzed with CTAn software (version 1.6.0.0, Bruker microCT, Kontich, Belgium) to assess bone parameters. (**C**) The decalcified femurs of rats were stained with TRAP and H&E. The number of TRAP^+^ osteoclasts was counted. Scale bar = 100 μm. Blue dots represent individual values for each replicate within the group. *** *p* < 0.001 vs. sham, ^##^ *p* < 0.05, and ^###^ *p* < 0.001 vs. OVX. *n* = 6. NS, not significant.

We used an OVX animal model to assess whether CRW prevents bone destruction in vivo. We collected the femurs of rats 12 weeks after the induction of the OVX and subjected them to micro-CT. Based on the micro-CT 3D images of the right femur, we determined that rats in the OVX group exhibited substantially higher trabecular bone loss compared to those in the sham group (Figure 7A). Conversely, OXV-mediated trabecular bone loss was noticeably decreased in rats orally treated with 100 mg/kg CRW [26,27] and 100 µg/kg E2 (17β-estradiol) [28,29] (Figure 7A). We further analyzed the obtained micro-CT results to assess the BMD and the trabecular parameters in the right femur using the CTAn software (version 1.6.0.0, Bruker microCT, Kontich, Belgium) program.

As summarized in Figure 7B, OVX-rat group lowered BMD, bone volume/tissue volume (BV/TV), and trabecular number (Tb. N) and increased trabecular separation (Tb. Sp) relative to sham group. However, we observed a significant restoration of the BMD, BV/TV, and Tb. N in the OVX/CRW group (Figure 7B). The Tb. Sp tended to decrease, although no significant difference was confirmed in the OVX/CRW group compared to the OVX group (Figure 7B). Trabecular thickness (Tb. Th) was reduced by OVX and trended higher in the OVX/CRW group than OVX, but the change was not significant (Figure 7B). In the OVX/E2 group used as a positive control, it was confirmed that BMD, BV/TV, and Tb. N—which dropped due to OVX and Tb. Sp, which was augmented due to OVX—were significantly recovered (Figure 7B). H&E staining revealed a decreased trabecular bone area in the OVX group compared with the sham group, whereas CRW treatment increased trabecular bone area in OVX mice. Compared with the sham group, the OVX group demonstrated an increase in the number of TRAP^+^ osteoclasts, whereas this increase was reduced in the OVX/CRW group (Figure 7C). Furthermore, the OVX-driven increase in TRAP-positive osteoclasts was reduced by CRW, with similar effects observed in the E2 group (Figure 7C).

## 3. Discussion

Osteoporosis causes progressive loss of bone mass and strength and raises fracture risk [30]. The clinical treatment of osteoporosis commonly involves using antiresorptive drugs, such as bisphosphonates and denosumab, which effectively reduce bone loss and fracture risk. However, despite their efficacy, serious side effects and potency reduction might limit their long-term usage [30,31,32]. These drugs have been associated with various adverse effects, including gastrointestinal problems, osteonecrosis of the jaw (ONJ), hypocalcemia, and atypical fractures. Hence, the development of alternative therapeutic schemes is required to mitigate the diverse adverse effects of current treatments [33].

Herbal medicines can be obtained from multiple parts of plants, including leaves, flowers, stems, roots, and seeds. Their global use has been steadily rising, and according to the World Health Organization (WHO), approximately 60% of the world’s population depends on herbal medicine for primary healthcare needs. Furthermore, the global trade in medicinal plants, raw herbal materials, and herbal preparations-derived products is reported to be growing by nearly 15% each year [34,35]. Herbal medicines are generally perceived as effective and relatively safe while minimizing adverse effects, leading to increasing interest in their application for various diseases.

In this study, we focused on *Coptidis rhizoma* (CR), a traditional herbal medicine widely prescribed in East Asia. CR contains high levels of isoquinoline alkaloids, most notably berberine, palmatine, coptisine, and epiberberine, with coptisine and berberine recognized as its principal bioactive components [20]. These compounds have been extensively studied for diverse pharmacological actions, including anti-inflammatory, antimicrobial, anti-cancer, and antioxidant activities, as well as for their therapeutic potential in metabolic, cardiovascular, and gastrointestinal disorders [20]. Previous studies have shown that coptisine restricts RANKL-driven osteoclastogenesis by limiting NF-κB and NFATc1 signaling, and that berberine reduces osteoclast differentiation by dampening Akt and MAPK pathways, which lowers TRAP^+^ MNCs and bone resorption [21,22]. Other natural products also curb osteoclast activity and preserve bone. Corylin reduces NFATc1 and c-Fos and lowers N.Oc/BS and Oc.S/BS in ovariectomized models [36]. Micheliolide lessens estrogen deficiency related bone loss mainly by decreasing osteoclast surface and number [37]. Icaritin suppresses RANKL-driven osteoclastogenesis and mitigates bone loss [38]. These reports support the view that CRW is a promising natural product candidate for osteoporosis therapy.

Based on the known inhibitory effects of coptisine and berberine, both of which are contained in CR, on osteoclast function and differentiation, we explored in this research the anti-osteoclastogenetic effect of CR. We initially conducted HPLC analysis to confirm the presence of coptisine and berberine in CRW. Our analysis confirmed that coptisine and berberine are the main constituents of CRW.

Osteoclast precursor cells of the monocyte–macrophage lineage fuse to generate TRAP^+^ MNCs, and TRAP staining as well as TRAP enzymatic readouts are standard indices of osteoclast formation and function [39,40]. Hence, TRAP staining and TRAP activity assays are commonly used methods for identifying and evaluating osteoclast activity. In this study, CRW reduced both the size and number of TRAP^+^ MNCs and lowered total TRAP activity in a concentration-dependent manner. Mature osteoclasts assemble an F-actin ring at the sealing zone where the cell contacts bone, which enables acidification and local proteolysis that together create resorption pits [23,39]. The osteoclast membrane differentiates into a ruffled border in the sealing zone, where it produces hydrochloric acid and several proteases, including MMP-9, MMP-14, and cathepsin K. These enzymes, along with the acidic environment, are crucial for the breakdown of both the bone matrix and hydroxyapatite crystals. The acidic environment dissolves the mineral component, while the proteases degrade the organic matrix, leading to the formation of a bone resorption pit. This coordinated action of acid production and enzymatic activity is essential for effective bone resorption [23]. Our study demonstrated that CRW blocks F-actin ring formation and decreases pit area on mineral surfaces, indicating attenuation of bone resorptive capacity.

Osteoclast differentiation is strictly controlled by several signaling molecules and factors, including two key cytokines, M-CSF and RANKL. M-CSF supports the survival and proliferation of osteoclast precursor cells, while RANKL promotes osteoclast differentiation [8]. Activation of the RANK/RANKL pathway initiates downstream signaling cascades, including the Akt, NF-κB, and MAPK pathways, during the early phase of osteoclast differentiation [41,42]. This cascade subsequently promotes the expression of transcription factors, including NF-κB, NFATc1, and AP-1 (c-Fos and c-Jun), which are crucial for osteoclast differentiation and formation. NF-κB is crucial for osteoclastogenesis, as evidenced in NF-κB-knockout mice lacking both NF-κB1 and NF-κB2, which exhibit severe defects in osteoclast function and formation, leading to the development of osteopetrosis [43,44,45]. NF-κB proteins are typically found in an inactive form in the cytoplasm, bound to IκBs. The primary heterodimeric form of NF-κB comprises p65 and p50 subunits [46]. Stimulation by RANKL leads to the degradation of IκB, resulting in the release of the p65 subunit of NF-κB [46]. The phosphorylated p65 subunit is then translocated to the nucleus, where it upregulates NFATc1 expression [39]. In this study, CRW suppressed the RANKL-activated phosphorylation of Akt, p38, ERK, JNK, and NF-κB p65, while also decreasing the nuclear translocation of NF-κB p65. When compared with previous findings, coptisine and berberine have been consistently reported to inhibit NF-κB signaling, whereas their effects on MAPK pathways were variable and context-dependent [21,22,47,48]. In contrast, our study demonstrated that CRW, containing both compounds, simultaneously suppressed MAPK, Akt, and NF-κB signaling during RANKL-induced osteoclast differentiation. Together, these results suggest the possibility that CRW, with its multiple bioactive constituents, may exert broader mechanistic regulation than individual compounds.

Both c-Fos and NFATc1 are key transcriptional regulators involved in regulating the osteoclast differentiation process and modulating the production of osteoclast-related marker genes [49]. c-Fos is an early determinant of osteoclast differentiation and positively regulates NFATc1 from an upstream position [50]. In osteoclast precursors, NFATc1 resides in the cytoplasm in a phosphorylated inactive state and, upon RANKL stimulation, becomes dephosphorylated and translocates to the nucleus [39]. Once in the nucleus, NFATc1 activates an osteoclast gene program that includes *OSCAR*, *ATP6V0D2*, *ACP5* (TRAP), *OC-STAMP*, *DC-STAMP*, *CTSK*, *CALCR*, and *MMP-9*, supporting osteoclast differentiation, fusion, and bone resorption [23,39]. Our results suggest that CRW efficiently diminished the activation of c-Fos and NFATc1 and consequently downregulated these osteoclast markers during RANKL-mediated osteoclast differentiation.

Although CRW contains multiple bioactive constituents, including berberine and coptisine, our study did not directly assess additive or synergistic interactions among these compounds. Rather, we intended to suggest the possibility that such interactions may contribute to the observed biological activity, in accordance with the multi-component and multi-target nature of herbal medicine. For example, the concentration of berberine in CRW at 25 μg/mL was calculated to be approximately 0.6525 μg/mL (about 1.75 μM), which falls within the effective range (0.1–5 μM) reported in previous studies using purified berberine [21]. This finding supports the plausibility that the overall inhibitory effects of CRW on osteoclast differentiation may arise not only from individual constituents but also from potential complementary or synergistic actions among them. Given that bone remodeling is governed by a balance between osteoclasts and osteoblasts, bone formation and osteoblast function are equally important for bone homeostasis. We did not evaluate osteoblasts in this work. Berberine, a principal constituent of CRW, has been reported to stimulate osteoblast differentiation and enhance bone formation through Runx2 and p38 MAPK signaling with increased expression of osteogenic markers including osteopontin and osteocalcin [51]. These findings suggest that CRW, which contains berberine as a major constituent, may also support osteoblast activity and bone formation. Future studies should determine whether CRW has dual effects on bone remodeling by inhibiting osteoclast activity and promoting osteoblast formation.

OVX animal models are commonly employed in studying osteoporosis and evaluating novel therapies for osteoporosis [52]. In our study, we confirmed that female SD rats with bone loss due to OVX surgery had decreased BMD and damaged trabecular bone structural properties. However, CRW prevented OVX-mediated bone loss and restored the structural properties of BV/TV, Tb. Th, Tb. N, and Tb. Sp. The positive control E2 produced similar improvements

In summary, CRW restrains RANKL-driven osteoclast differentiation and function by suppressing early kinase signaling and key transcription factors, with downstream reductions in osteoclast genes. CRW also improved OVX-associated bone loss in vivo. These findings support CRW as a potential treatment for disorders driven by excessive osteoclast activity.

## 4. Materials and Methods

### 4.1. Animals and Reagents

Six-week-old male ICR mice and seven-week-old female Sprague Dawley rats were purchased from Samtako Bio Korea (Osan, Republic of Korea). Berberine was sourced from MilliporeSigma (Darmstadt, Germany) and coptisine from Chengdu Biopurify Phytochemicals Ltd. (Chengdu, China). Recombinant human RANKL and M-CSF were purchased from PeproTech EC Ltd. (London, UK). The following primary antibodies were used: NFATc1 (1:1000, SC-7294), c-Fos (1:1000, SC-7202), β-actin (1:1000, SC-47778), and Lamin B (1:1000, SC-6216) were from Santa Cruz Biotechnology Inc. (Santa Cruz, CA, USA) and from Cell Signaling Technology (Beverly, MA, USA); antibodies against total proteins Akt (9272), p38 (9212), JNK (9252), ERK (9102), an IκB (4812), and NF-κB p65 (8242), as well as their phospho-specific counterparts p-Akt (9271), p-p38 (9211), p-JNK (9251), p-IκB (2859), p-NF-κB p65 (3033), and p-ERK (4370) at 1:1000 dilution. HRP-conjugated secondary antibodies were anti-mouse IgG (1:5000, SC-2005), and anti-rabbit IgG (1:5000, SC-2004) from Santa Cruz Biotechnology. 17β-estradiol (E2) was purchased from MilliporeSigma.

### 4.2. Ethics Statement

All animal procedures were approved by the Institutional Animal Care and Use Committee of Wonkwang University (WKU20-21). Animals were maintained in a laminar air-flow room facility at 22 ± 1 °C and 55 ± 5% relative humidity with a 12 h light and 12 h dark cycle.

### 4.3. High-Performance Liquid Chromatography (HPLC) Analysis

Analyses were performed on an Agilent 1200 system comprising a quaternary pump, autosampler, column oven, and variable wavelength detector. Separation used a Zorbax SB-C18 column (4.6 × 150 mm, 4.0 µm, Agilent Technologies, Santa Clara, CA, USA). The detailed gradient, mobile phase, flow rate, temperature, and detection wavelength are summarized in Table 1.

### 4.4. Preparation of Coptidis Rhizoma Water Extract (CRW)

Crude *Coptidis rhizoma* (CR, *Coptis chinensis* Franch., *C. deltoidea* C. Y. Cheng et Hsiao or *C. teeta* Wall) was procured from Humanherb (Daegu, Republic of Korea) and manufactured under KFDA-certified GMP. CR used in this study are 5-year, and 6-year CR, and the harvest time was November to December. A voucher specimen (WP-2019-02) was deposited at the College of Pharmacy, Wonkwang University. Coarsely cut CR (100 g) was immersed in distilled water for 30 min, and then its contents were extracted by boiling at 100 °C for 2 h. The boiled extracts were percolated through 110 mm Advantec No. 2 filter paper (Advantec, Tokyo, Japan). The filtrate was concentrated by rotary evaporator and lyophilized with a Bondiro freeze dryer (Ilshin BioBase Co., Ltd., Dongducheon, Republic of Korea). The dry extract yield was 45.74 g (45.74%). Powders were stored at −20 °C until use and archived at the College of Pharmacy for future studies.

### 4.5. Isolation of Bone Marrow Macrophages (BMMs)

Bone marrow cells (BMCs) were collected from femurs and tibiae of 5-week-old male ICR mice as previously described [53]. BMCs were first cultured for 24 h in growth medium (GM) containing M-CSF (10 ng/mL). Nonadherent cells were then replated with M-CSF (30 ng/mL) for 3 days, after which attached cells were considered BMMs. All cultures were maintained at 37 °C in a humidified incubator with 5% CO_2_.

### 4.6. Cell Viability Assay

BMMs were seeded at 1 × 10^4^ cells per well and treated with CRW at 1, 5, or 25 µg/mL in GM supplemented with M-CSF (30 ng/mL) for 4 days (*n* = 4). Medium was refreshed on day 3. Cell viability was measured using Cell Proliferation Kit II (XTT) (Roche, Mannheim, Germany) after 4 h incubation at 37 °C, and absorbance at 450 nm was read on a Thermomax microplate reader (Molecular Devices, Sunnyvale, CA, USA).

### 4.7. Osteoclast Differentiation and TRAP Assay

To induce osteoclastogenesis, BMMs were cultured with M-CSF (30 ng/mL) and RANKL (100 ng/mL) in the presence of CRW (1, 5, or 25 µg/mL) for 4 days. Cells were fixed with 4% paraformaldehyde (T&I, Chuncheon, Republic of Korea) for 10 min, permeabilized with 0.2% Triton X-100 for 10 min, and stained in TRAP buffer containing Fast red violet LB 1 mg/mL (MilliporeSigma, Darmstadt, Germany), naphthol AS-MX phosphate 100 µg/mL (MilliporeSigma, Darmstadt, Germany), 0.1 M sodium acetate pH 5.0, and 50 mM sodium tartrate. TRAP^+^ MNCs with at least three nuclei were counted as mature osteoclasts (*n* = 3). Representative images were acquired using a Leica microscope (Leica Microsystems, Wetzlar, Germany). For TRAP activity, lysates were prepared in 1% Triton X-100 in TRAP assay buffer, incubated with p-nitrophenyl phosphate 1 mg/mL at 37 °C for 10 min; the reaction was stopped with 1 M NaOH, and absorbance was recorded at 405 nm (*n* = 3).

### 4.8. F-Acting Ring Staining

BMMs cultured with M-CSF (30 ng/mL) and RANKL (100 ng/mL) were treated with CRW (1, 5, and 25 µg/mL) (*n* = 3). After fixation with 4% paraformaldehyde and permeabilized with 0.1% Triton X-100, cells were stained with phalloidin (1:200, Thermo Fisher Scientific, Waltham, MA, USA) for 30 min to visualize the actin cytoskeleton. Nuclei were counterstained with DAPI (Sigma-Aldrich, St. Louis, MO, USA). Images were obtained on an EVOS FL fluorescence microscope (Thermo Fisher Scientific, Waltham, MA, USA).

### 4.9. Western Blotting

Cells were lysed in RIPA buffer (Bio-sesang, Seongnam, Republic of Korea) containing protease inhibitors (MilliporeSigma) and sodium orthovanadate (Na_3_VO_4_, MilliporeSigma). Following the manufacturer’s protocol, the cytoplasmic and nuclear protein fractions were prepared using the NE-PER Nuclear and Cytoplasmic Extraction Kit (Thermo Fisher Scientific, Inc.). Equal protein amounts were separated by 10% SDS-PAGE and transferred to PVDF membranes (Bio-Rad, Hercules, CA, USA). After blocking with 5% BSA (MilliporeSigma, Darmstadt, Germany) for 1 h, membranes were incubated with primary antibodies (1:1000) at 4 °C overnight, washed with TBST, and probed with HRP-conjugated secondary antibodies (1:5000) for 1 h. Bands were visualized using Western ECL solution (Bio-Rad) on a FlourChem E system (Cell Bioscience, Santa Clara, CA, USA).

### 4.10. Quantitative qPCR

Total RNA was isolated using NucleoZOL (Macherey-Nagel, Dueren, Germany) and quantified with a NanoDrop2000 (Thermo Fisher Scientific, Waltham, MA, USA). cDNA was synthesized with the AMPIGENE cDNA Synthesis Kit (Enzo Life Sciences, Farmingdale, NY, USA). qPCR was performed using AMPIGENE qPCR Green Mix Hi-Rox (Enzo Life Sciences, Farmingdale, NY, USA) on a StepOnePlus system (Applied Biosystems, Carlsbad, CA, USA) following the manufacturer’s instructions. The target genes were amplified using the primers indicated in Table 2. Relative transcript levels were normalized to mouse GAPDH and calculated by the 2^−ΔΔCq^ method [54].

### 4.11. Immunocytochemistry

BMMs were fixed with 4% paraformaldehyde and permeabilized using 0.1% Triton X-100 (MP Biomedicals, Solon, OH, USA). Following a block with 5% BSA, attached cells were exposed to anti-NFATc1 and NF-κB antibodies (1:500). Following PBS washes, Alexa fluor 488 secondary antibodies were applied at 1:200 (anti-mouse A32723 and anti-rabbit A11008; Thermo Fisher Scientific, Waltham, MA, USA), and nuclei were counterstained with DAPI (D9542, Sigma-Aldrich, St. Louis, MO, USA). Images were performed using an EVOS FL fluorescence microscope (Thermo Fisher Scientific, Waltham, MA, USA). Cell fluorescence was measured using ImageJ (version 1.54g, NIH, Bethesda, MD, USA), and NFATc1 and NF-κB p65 nuclear translocation was quantified by measuring the area and fluorescence intensity of nuclear and total NFATc1 and NF-κB p65 (*n* = 3).

### 4.12. Bone Resorption Assay

Primary osteoblasts were isolated from calvariae of 1-day-old ICR mice [53]. Co-cultures of primary osteoblasts and BMCs were established on collagen gel-coated dishes (Corning Inc., Corning, NY, USA) in α-MEM containing 10^−8^ M 1,25-dihydroxyvitamin D3 (VitD3) and 10^−6^ M prostaglandin E2 (PGE2) for 1 week. Osteoclasts obtained from co-cultures were detached with 0.1% collagenase and replated on Osteo assay surface plates (Corning Inc., Corning, NY, USA) with or without CRW in M-CSF (30 ng/mL) and RANKL (100 ng/mL) [53]. After removing cells with 10% NaClO, resorption pits were imaged microscopically and quantified using ImageJ (version 1.54g, NIH, Bethesda, MD, USA) (*n* = 3).

### 4.13. Animal Bone-Loss Model

Female SD rats (weighing 130–170 g) were subjected to sham surgery or bilateral ovariectomy under ketamine-xylazine anesthesia. Animals were randomly assigned to four groups (*n* = 6 rats/group): (1) sham-administered PBS, (2) OVX-administered PBS, (3) OVX treated with E2 (100 µg/kg, subcutaneously, S.C., E2758, Sigma-Aldrich, St. Louis, MO, USA), (4) OVX-administered CRW (100 mg/kg, orally, P.O.). Following a one-week recovery, PBS or CRW were orally administered once daily for 12 weeks. After the final dose and 24 h fast, animals were euthanized, and femurs were collected and fixed in 4% paraformaldehyde for 24 h. E2 was employed as a positive control for estrogen replacement.

### 4.14. Micro-CT and Histology

Micro-CT imaging was performed with a Skyscan 1076 system (Bruker, Kontich, Belgium) following Han et al. [53]. After scanning, femurs were decalcified in 12% EDTA at pH 7.4 for 3 weeks, embedded in paraffin, sectioned at 5 µm with a Leica RM2125 microtome (Leica Microsystems, Bannockburn, IL, USA), and stained with H&E and TRAP.

### 4.15. Statistical Analysis

Data are reported as mean ± standard deviation (SD) from at least three independent experiments. Group differences were assessed by one-way ANOVA in the SPSS version 12.0 (Korean edition; SPSS Inc., Chicago, IL, USA), followed by Tukey’s HSD post hoc test. Statistical significance was set at *p* < 0.05.

## Figures and Tables

**Table 1:** The condition of analysis of CRW by HPLC system.

Parameters	Condition		
Instrument	Agilent 1200 series HPLC system (Agilent Technologies, Santa Clara, CA, USA)		
Column	C18 4.6 mm × 150 mm, 4.0 μm		
Column temp.	30 °C		
Mobile phase	Time (min)	A (%)	B (%)
	0	85	15
	20	60	40
	A: 0.5% formic acid in DW (*v*/*v*, %) B: 0.5% formic acid in ACN:MeOH (3:7) (*v*/*v*, %)		
Detector	Waters 996 Photodiode Array Detector 280 nm		
Flow rate	0.5 mL/min		
Injection volume	10 μL		
Run time	35min		

**Table 2:** Primer sequences uesd for qPCR.

Target Gene	Gene Accession Number		Primer Sequence (5′-3′)
*c-Fos*	NM_010234.3	Forward	CTGGTGCAGCCCACTCTGGTC
		Reverse	CTTTCAGCAGATTGGCAATCTC
*NFATc1*	NM_198429.2	Forward	CAACGCCCTGACCACCGATAG
		Reverse	GGCTGCCTTCCGTCTCATAGT
*ACP5*	NM_007388	Forward	ACTTCCCCAGCCCTTACTAC
		Reverse	TCAGCACATAGCCCACACCG
*OSCAR*	NM_175632.3	Forward	CTGCTGGTAACGGATCAGCTCCCCAGA
		Reverse	CCAAGGAGCCAGAACCTTCGAAACT
*ATP6V0D2*	NM_175406.3	Forward	TCAGATCTCTTCAAGGCTGTGCTG
		Reverse	GTGCCAAATGAGTTCAGAGTGATG
*CTSK*	NM_007802.4	Forward	ACGGAGGCATTGACTCTGAAGATG
		Reverse	GTTGTTCTTATTCCGAGCCAAGAG
*MMP-9*	NM_013599.4	Forward	TCCAACCTCACGGACACCC
		Reverse	AGCAAAGCCGGCCGTAGA
*CALCR*	NM_001377018.1	Forward	TCCAACAAGGTGCTTGGGAA
		Reverse	CTTGAACTGCGTCCACTGGG
*DC-STAMP*	NM_001289506.1	Forward	TCCTCCATGAACAAACAGTTCCA
		Reverse	AGACGTGGTTTAGGAATGCAGCTC
*OC-STAMP*	NM_029021.1	Forward	ATGAGGACCATCAGGGCAGCCACG
		Reverse	GGAGAAGCTGGGTCAGTAGTTCGT
*GAPDH*	NM_001289726.1	Forward	ACCACAGTCCATGCCATCAC
		Reverse	TCCACCACCCTGTTGCTGTA

## Data Availability

The data presented in this study are available in this article.
